# Pyroptosis: A Novel Therapeutic Target for Bioactive Compounds in Human Disease Treatment? A Narrative Review

**DOI:** 10.3390/nu17030461

**Published:** 2025-01-27

**Authors:** Bei Yang, Zexiu Qi, Yasmany Armas Diaz, Manuela Cassotta, Giuseppe Grosso, Danila Cianciosi, Di Zhang, Xiaobo Zou, José L. Quiles, Maurizio Battino, Francesca Giampieri

**Affiliations:** 1 Joint Laboratory on Food Science, Nutrition, and Intelligent Processing of Foods, Polytechnic University of Marche, Italy, Universidad Europea del Atlántico Spain and Jiangsu University, China at Polytechnic University of Marche, 60130 Ancona, Italy; 2 Dipartimento di Scienze Cliniche Specialistiche e Odontostomatologiche, Università Politecnica delle Marche, Via Ranieri 65, 60130 Ancona, Italy; 3 Research Group on Food, Nutritional Biochemistry and Health, Universidad Europea del Atlántico, Isabel Torres 21, 39011 Santander, Spain; 4 Department of Biomedical and Biotechnological Sciences, University of Catania, 95123 Catania, Italy; 5 Center for Human Nutrition and Mediterranean Foods (NUTREA), University of Catania, 95123 Catania, Italy; 6 Joint Laboratory on Food Science, Nutrition, and Intelligent Processing of Foods, Polytechnic University of Marche, Italy, Universidad Europea del Atlántico Spain and Jiangsu University, China at Jiangsu University, Zhenjiang 212013, China; 7 School of Food and Biological Engineering, Jiangsu University, Zhenjiang 212013, China; 8 Department of Physiology, Institute of Nutrition and Food Technology “Jose Mataix”, Biomedical Research Center, University of Granada, 18016 Granada, Spain; 9 International Joint Research Laboratory of Intelligent Agriculture and Agri-Products Processing, Jiangsu University, Zhenjiang 212013, China; 10 International Research Center for Food Nutrition and Safety, Jiangsu University, Zhenjiang 212013, China

**Keywords:** pyroptosis, dietary bioactive compounds, inflammation, inflammasome, human disease treatment

## Abstract

**Background/Objectives**: Bioactive compounds possess the ability to maintain health and improve diseases by regulating inflammation and cell death processes. Pyroptosis is programmed cell death related to inflammation and exerts a critical role in the development and progression of different types of diseases. This narrative review aims to investigate and discuss the effects of dietary bioactive compounds on pyroptosis in different common human pathologies, such as inflammatory disease, bacterial infection, injury disease, cancer, diabetes and heart disease, etc. **Method**: Studies published in the major databases until December 2024 in English were considered, for a total of 50 papers. **Results**: The current evidence demonstrated that the bioactive compounds are able to regulate the pyroptosis process by modulating different inflammasome sensors (NLRP1, NLRP3, and AIM2), caspase family proteins (caspase-1, caspase-3, and caspase-11), and gasdermins (GSDMD and GSDME) in many pathological conditions related to inflammation, including cancer and cardiovascular diseases. **Conclusions**: Bioactive compounds have powerful potential to be the candidate drug for pyroptosis modulation in inflammatory diseases, even if more clinical studies are needed to confirm the effects and establish efficient doses for humans.

## 1. Introduction

Bioactive compounds are commonly contained in many foods and medical herbs. Numerous studies have demonstrated the importance of bioactive compounds in human health, attributed to their antioxidant, anti-inflammation, anti-diabetic, anti-bacteria, hepatoprotective, hypoglycemic, immunomodulatory, wound healing, cardioprotective, and neuro protective properties [1,2,3,4,5,6,7,8]. Indeed, bioactive compounds have great potential in disease treatment: noteworthy, 47% of anticancer drugs are of natural origin or directly derive from nature, which indicates that the bioactive compounds may contribute not only to the prevention of human diseases, but also to their treatment [9].

**Figure 1:**
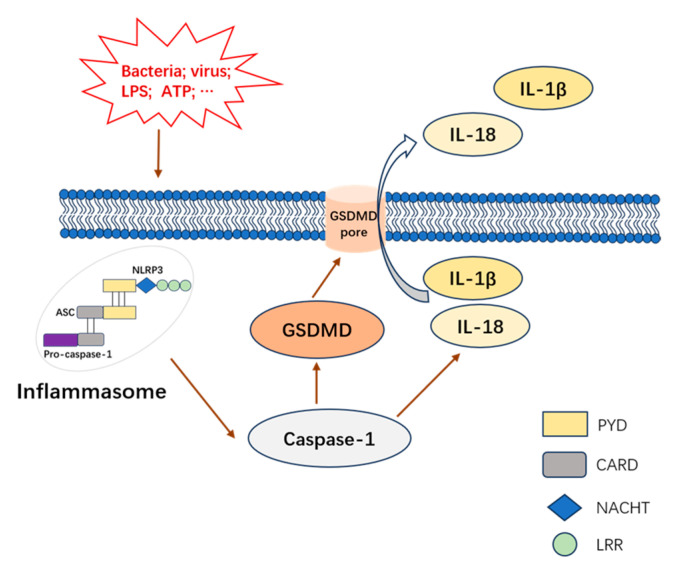
Mechanism of canonical pyroptosis.

Pyroptosis, inflammatory programmed cell death, has become increasingly popular among scientists for its influence on innate immunity and diseases [10]. The beginning of pyroptosis is caused by the inflammasome, usually formed with the combination of nucleotide-binding oligomerization domain-like receptors (NLRs) synthesized from two parts (a leucine-rich repeat (LRR) and nucleotide-binding oligomerization domain (NACHT)), an adapter apoptosis-associated speck-like protein containing a caspase recruitment domain (CARD) (ASC), and pro-caspase-1, which lead to the activation of inflammatory caspase-1 [10,11]. Gasdermin D (GSDMD) is cleaved as soon as caspase-1 is provoked, following the disruption of membrane integrity by oligomerized N-terminal domain-induced membrane pores [10,12]. The whole process, which includes inflammasome assembly, the activation of caspase-1, GSDMD cleavage and the excretion of IL-1β (Interleukin-1β) and Interleukin-18 (IL-18), is regarded as canonical pyroptotic death [10,13,14,15,16]. NLR pyrin domain-containing (PYD) 3 (NLRP3) is the most common inflammasome sensor leading to canonical pyroptotic death (Figure 1).

On the contrary, in the noncanonical pathway, intracellular caspase-4/5 or caspase-11 is first activated by lipopolysaccharides (LPS), which then mediate the cleavage of GSDMD rather than pro-IL-1β/pro-IL-18, but these caspases can indirectly activate the NLRP3 inflammasome/caspase-1 pathway and induce the secretion of IL-1β and IL-18 [10,17]. In addition, there are some other pathways directly involved in the pyroptosis process, such as the caspase-3/8-medicated pathway [18,19,20,21] and granzyme-medicated pathway [22,23,24].

Pyroptosis is closely related to human diseases [25], while few researchers concentrate on reviewing the significance of bioactive compounds on them. This narrative review focuses on the role of pyroptosis in the prevention and therapeutic processes regulated by bioactive compounds, investigating the potential value of pyroptosis in improving the therapeutic efficacy of bioactive compounds.

## 2. Bibliographic Search

Studies published in the major databases (Pubmed, Scopus, and Web of Sciences) until December 2024 in English were considered using specific keywords like “bioactive compounds”, “pyroptosis”, and “specific disease” (i.e., inflammatory diseases, bacterial infection, cancer…). This initial research found 1470 potentially relevant publications, which became 50 after excluding unrelated studies (out of scope) or duplicates.

## 3. Bioactive Compounds Regulate Pyroptosis in Inflammatory Disease

The excretion of pro-inflammatory factors is the most essential process in the immune response and plays a critical role in numerous inflammatory pathologies [26]. Bioactive compounds have shown anti-inflammation properties by regulating a variety of cellular pathways, and their importance in pyroptosis is worth exploring and explaining well.

Many studies have evaluated the effects of bioactive compounds on pyroptosis by using macrophages, since they provide an essential and efficient cell model for research (Table 1).

For example, 1′-Acetoxychavicol acetate (ACA), from the rhizome of the tropical ginger *Alpinia* species, may avoid inflammasome activation and caspase-1 and IL-1β production, finally inhibiting the cleavage of GSDMD in mouse bone-marrow-derived macrophages (BMMs) [27]. In addition, in LPS-stressed mouse macrophages’ cell line J774A.1, Toonaone D [28] from *Toona ciliate* ameliorated pyroptosis by blocking the activation of NLRP3 inflammasome, IL-1β, and caspase-1. Similarly, celastrol from Tripterygium wilfordii Hook [26] and Punicalin found a decrease in the excretion of IL-18 and IL-1β in pomegranate by suppressing the levels of NLRP3 and caspase-1 in LPS/ATP-induced J774A.1 cells; these results were ascribed to the decrease in reactive oxygen species (ROS) production [29]. Additionally, celastrol also reduced NF-κB activation, while Punicalin blocked the production of ASC and GSDMD-N. Moreover, Ye et al. found that scutellarin, derived from *Erigeron breviscapus*, can reduce the release of caspase-1 and caspase-11, the secretion of IL-1β, the activation of NLRP3 inflammasome, and the formation of ASC specks in both J774A.1 and bone-marrow-derived macrophages’ (BMDMs) cell lines [30]. Interestingly, the scutellarin-mediated suppression of caspase-11 did not depend on the ASC/NLRP3 inflammasome pathway, which was also found in the RAW 264.7 macrophage cell line that lacked ASC expression but depended on the cyclic adenosine monophosphate (cAMP)/protein kinase A (PKA) pathway [30]. Another component of the *Panax japonicus* rhizome, named Chikusetsu saponin IVa (CS), can decline the NLRP3 inflammasome assembly in BMDMs macrophages by constraining the nuclear translocation of nuclear factor kappa-B (NF-κB) p65, suppressing the expression of prostaglandin-endoperoxide synthase 2 (ptgs2) and nitrite oxide synthase-2 (NOS-2) in LPS-induced cells [31]. However, the efficiency dose of bioactive compounds in therapy is not like “give? more, get more” even though most of them can inhibit pyroptosis in a dose-dependent way. For example, Srikanth et al. found that low concentrations of docosahexaenoic acid (DHA) (30 μM) attenuated intracellular inflammatory factor expression, while higher concentrations (200 μM) induced the cleavage of pro-caspase-1 and promoted the excretion of IL-1β, indicating that cells underwent a proinflammatory cell death program as pyroptosis [32].

**Table 1:** Effects of bioactive compounds on inflammatory disease.

Bioactive Compound	Derived from	In Vivo/Vitro	Dose	Effect	Ref.
1′-Acetoxychavicol acetate	*Alpinia*	In vitro	0.1, 1, 2.5 μM	Inhibition of mitochondrial ROS generation Prevention of oxidized mitochondrial DNA release Decrease in NLRP3, caspase-1, ASC, IL-1β, and GSDMD cleavage	[27]
		In vivo	1.5 mg/kg	Decrease in NLRP3 and caspase-1	
Celastrol	Tripterygium wilfordii *Hook*	In vitro	12.5, 25, 50 nM	Reduction in IL-1β and IL-18, NLRP3 inflammasome, caspase-1, LDH leakage Decrease in ROS and NF-κB	[26]
DHA	Food	In vitro	30 µM	Suppression of IL-1β	[32]
			200 µM	Increase in pyroptosis Increase in IL-1β, caspase-1	
Punicalin	Leaves of *Terminalia catappa* L and pomegranate husk	In vitro	20, 50, 100 µM	Decrease in ROS Decrease in NLRP3, caspase 1, ASC and GSDMD-N, IL-1β, and IL-18	[29]
Resveratrol	Grapes, peanuts, and *Reynoutria japonica*	In vitro	7.5, 15, 30 µM	Reduction in caspase-1, NLRP3, IL-1β, and IL-18 Increase in mitochondrial membrane potential Inhibition of P62 and Pink1 Increase in TOMM20, Parkin, and LC3B-II Increase in mitophagy	[33]
		In vivo	15 mg/kg	Improvement of GA Improvement of peritonitis Decrease in IL-1β and IL-18	
Scutellarin	*Erigeron breviscapus*	In vitro	12.5, 25, 50 µM	Inhibition of caspase-11p26 and GSDMD-NT Reduction in pyroptosis. Suppression of NLRP3, ASC, IL-1β, and caspase-1p10 Increase in Ser/Thr phosphorylation of PKA	[30]
		In vivo	50, 100 mg/kg	Decrease in IL-1β	
Toonaones D, limonoids	*Toona ciliata* M. Roem.	In vitro	2.5, 5, 10 µM	Inhibition of GMDMD, caspase-1, and IL-1β	[28]
Chikusetsu saponin IVa	*Panax japonicus*	In vitro	10, 20, 40 µM	Inhibition of NLRP3, ASC, caspase-1, and IL-1β Decrease in LDH release Suppression of NF-κB	[31]
		In vivo	50, 100 mg/kg	Improvement of lipid homeostasis Inhibition of inflammation in adipose tissue Decrease in chemokines and cytokines Inhibition of the accumulation of adipose tissue macrophages Decrease in IL-1β, caspase-1, NLRP3, and ASC Suppression of NF-κB	
Ellagic acid	*Galla chinensis* and *Pericarpium granati*	In vivo	10, 50 mg/kg	Decrease in TNFα, IL-6, IL-17A and IL-10, NLRP3, and caspase 1 Improvement of neuroinflammation, demyelination, and axonal damage Improvement of MBP, GFAP, and Iba1 immunoreactivity.	[34]
Shionone	*Aster tataricus*	In vitro	2.5, 5, 10 µg/mL	Reduction in ASC, pro-caspase-1, GSDMD, GSDMD-N, caspase-1, NF-κB, and NLRP3	[35]
		In vivo	50, 100 mg/kg	Improvement of bladder wet weight, score of hemorrhage and edema Reduction in ASC, pro-caspase-1, GSDMD, GSDMD-N, caspase-1, NF-κB, and NLRP3	

In vivo research is essential for providing a wide vision for exploring the anti-pyroptosis properties of bioactive compounds. In this context, ACA was able to prevent NLRP3 inflammasome activation in the monosodium urate (MSU) crystal-induced peritonitis and dextran sodium sulfate-induced colitis mouse models, accompanied by decreasing caspase-1 activation [27]. Another research showed that resveratrol significantly improved the synovitis and gait score of rats affected by gouty arthritis (GA) and attenuated the peritoneal inflammation caused by MSU in mice [33]. Furthermore, CS can improve high-fat-diet (HFD)-induced lipid accumulation and inhibit inflammation in adipose tissue, (i) suppressing the levels of inflammatory factors and the secretion of the chemokines/cytokines, (ii) blocking the recruitment of adipose tissue macrophages (ATMs), shifting their polarization from M1 to M2, (iii) inhibiting the NLRP3 inflammasome combination, and (iv) blocking caspase-1 and IL-1β activation in mice [31]. Additionally, ellagic acid alleviated neuroinflammation, axonal damage, and demyelination in spinal cord specimens of the experimental autoimmune encephalomyelitis (EAE) group by decreasing the tissue NLRP3 inflammasome, increasing the expression of the myelin basic protein (MBP), and blocking the immunoreactivity of the glial fibrillary acidic protein (GFAP) and ionized calcium-binding adaptor molecule 1 (Iba1) [34]. Finally, shionone was isolated from the herbal *Aster tataricus*, and significantly declined the bladder wet weight and the score of edema and hemorrhage by reducing the expression of NF-κB and pyroptosis protein (ASC, caspase-1, GSDMD-N, and NLRP3,) in interstitial cystitis (IC) rats [35].

## 4. Bioactive Compounds Regulate Pyroptosis in Bacterial Infection

Bacterial infection occurs frequently in clinical surgery and daily life and may cause an adverse reaction to wound prognosis [36]. Many studies have demonstrated that the regulation of inflammasome activation and the pyroptosis process can provide protection against bacterial infection (Table 2) [37,38].

Natural dietary products possess numerous different chemical structures and many special biological properties that also include the inhibition of bacterial growth [39]. For example, the essential oil of Artemisia argyi H. Lév. and Vaniot (EOAA), a traditional medicine, can inhibit bacterial infection by regulating pyroptosis: specifically, EOAA blocked the assembly of NLRP3 inflammasome, the cleavage of pro-caspase-1 and pro-IL-1β, and the phosphorylation and translocation of NF-κB p65 in an MSU- and nigericin-induced bacterial infection cell model [40].

LPS activates inflammatory caspase-11 and leads to pyroptosis, providing a new possibility against bacterial infection. A recent study reported that scutellarin inhibited the LPS-induced generation of caspase-11p26, the cleavage of GSDMD-NT, and the formation of ASC speck, leading to reduced pyroptosis; interestingly, the Ser/Thr phosphorylation of caspase-11 at PKA-specific sites was activated by scutellarin, which suggested that this compound suppressed caspase-11 and pyroptosis by partly modulating the PKA signaling pathway in macrophages [41]. Similarly, Yang et al. found that Taraxasterol (TAS) suppressed the production of caspase-1, the secretion of IL-1β, GSDMD cleavage, and ASC speck formation in LPS-induced macrophages, and further proved that TAS prevented the assembly of the NLRP3 inflammasome by decreasing the expression of the mechanistic target of rapamycin (mTOR) complex 1 (mTORC1) and mTORC2 [42].

High-mobility group box 1 (HMGB1), as a nuclear protein, exerts plenty of functions in the nucleus of all mammalian cells. Different viral and bacterial infections can promote the release of HMGB1 and can activate pro-inflammatory cytokines from immune cells by modulating the RAGE, TLR2/TLR, and AMP-activated protein kinase (AMPK) pathway [43,44]. Piperine, a natural compound of black pepper (*Piper nigrum* Linn), was found to relieve LPS/ATP-induced pyroptosis and suppress IL-1β and HMGB1 by inhibiting AMPK activation [38]. In addition, one comparative study has reported that schisandrin A, B, and C were able to suppress *Propionibacterium acnes*-induced pyroptosis by suppressing NLRP3 inflammasome activation, declining the production of mitochondrial ROS, ATP release, and IL-1β secretion [45]. Further in vivo studies reported that piperine [38], scutellarin [41], and taraxasterol [42] reduced serum IL-1β levels of *Eschirichia coli*-infected mice, proving that bioactive compounds performed an antibacterial function by regulating pyroptosis.

**Table 2:** Effects of bioactive compounds on bacterial infection.

Bioactive Compound	Derived from	In Vivo/Vitro	Dose	Effect	Ref.
Oil of *Artemisia argyi* H.Lév. and Vaniot	*Artemisia argyi* H. Lév. and Vaniot	In vitro	SDAO: 22.5, 33.75, 45 μg/mL SFEAO: 68, 102, 136 μg/ml	Inhibition of NLRP3, caspase-1, NF-κB, and IL-1β	[40]
Piperine	Black pepper (*Piper nigrum Linn*)	In vitro	40, 80, 160 μM	Induction of pyroptosis Suppression of IL-1β or HMGB1 Suppression of AMPK	[38]
		In vivo	20 mg/kg	Improvement of peritoneal Reduction in serum IL-1β	
Scutellarin	*Erigeron breviscapus*	In vitro	0.1, 0.2, 0.4 mM	Decrease in ASC, caspase-1, IL-1β, and NLRP3 PKA pathway activation	[41]
		In vivo	100, 200 mg/kg	Improvement of survival mice Decrease in serum IL-1β	
Taraxasterol	Dandelion (*Taraxacum mongolicum Hand. -Mazz.*)	In vitro	25, 50, 100 μM	Decrease in ASC, GSDMD, IL-1β, and caspase-1 Suppression of mTORC1 and mTORC2	[42]
		In vivo	20 mg/kg	Improvement of survival mice Decrease in serum IL-1β	
Schisandrin A, B, and C	*Schisandra chinensis* (Turcz.) Baill.	In vitro	10 μM	Inhibition of NLRP3 inflammasome, IL-1β Decrease in mitochondrial ROS	[45]
Berberine	*Rhizoma coptidis* and *Cortex phellodendri*	In vitro	0.75, 1.5, 3.0 μM	Increase in caspase-1p10, IL-1β Activation of AMPK signal pathway	[46]
		In vivo	100 mg/kg	Decrease in peritoneal live bacterial Improvement of survival mice Increase in IL-1β Increase in neutrophil recruitment in the peritoneal cavity	

Another interesting study demonstrated that berberine increased pyroptosis by enhancing inflammasome activation, up-regulating the expression of caspase-1p10 and IL-1β in LPS/ATP-induced macrophages by activating the AMPK signal pathway; nevertheless, the peritoneal live bacterial load in vivo was remarkably decreased after treatment with berberine [46]. Berberine showed converse results in cell research (increased pyroptosis) compared with previous studies, but the same anti-bacterial effect may be due to the different cellular response to the inducer. Indeed, extracellular ATP can bind to its receptor P2X7R in the cell membrane and can promote the cleavage of caspase-1 and the assembly of NLRP3 inflammasomes, inducing IL-1β excretion [47], which in turn promotes bacterial killing by macrophages [48].

## 5. Bioactive Compounds Regulate Pyroptosis in Cancer

Pyroptosis also plays an essential role in tumor immunotherapy. Acute and chronic states of pyroptosis activate numerous immune cells, which not only promote massive cancer cell death, but also enhance anticancer immunity to suppress tumor growth [49,50]. A large number of natural compounds were reported to have anticancer potential by inducing cell pyroptosis (Table 3).

For example, in two studies on breast cancer, DHA induced the cleavage of pro-caspase-1 and GSDMD, increased the mature IL-1β and translocation of HMGB1, and accelerated the generation of membrane pores in breast cancer cells [51]; differently, dihydroartemisinin induced pyroptosis by cleaving gasdermin E (GSDME) through the activation of caspase-3 rather than caspase-1, as well as increasing those absent in melanoma 2 (AIM2), IL-1β, IL-18, and HMGB1 [52]. Pro-IL-1β/ IL-18 can only be cleaved by caspase-1; however, AIM2 can assemble inflammasomes and cleave pro-caspase-1 by its HIN-200 domain [10]. In addition, dihydroartemisinin prevented the breast xenograft tumors growth in BALB/c mice [52].

MiR-200b is one of the miRNAs with potential anticancer activity. A recent paper found that in miR-200b-transfected breast cancer cells, the levels of IL-1β, IL-18, ASC, caspase 1, and NLRP3 were firstly increased by miR-200b, then enhanced by nobiletin [53]. Similarly, Ding et al. highlighted that dioscin inhibited osteosarcoma (OS) proliferation in vivo and induced pyroptosis by increasing the activation of caspase-3 and the cleavage of GSDME-N in OS cells as well [54]. Another recent study demonstrated the anticancer ability of bioactive compounds by regulating pyroptosis in nasopharyngeal carcinoma: tanshinone IIA upregulated the concentration of cleaved GSDMD and caspase-1 and promoted the secretion of inflammatory cytokines including IL-18 and IL-1β in nasopharyngeal carcinoma HK1 cells [55]. Data about the regulation of pyroptosis in hepatocellular carcinoma by natural compounds are also published. For instance, Liang et al. reported that curcumin increased the cleaved GSDME N-terminus in HepG2 cells by means of up-regulating intracellular ROS levels [56]. Meanwhile, curcumin was found to have similar antitumor properties in malignant mesothelioma (MM) cells by increasing NLRP3 and caspase-1; unfortunately, curcumin treatment in vivo did not reduce the MM tumor burden [57]. Many contributions have been made by researchers who explored the pyroptotic effect in the anticancer capacity of bioactive compounds; however, more in vivo investigations are required to transform the evidence obtained in vitro into actual in vivo results and to comprehend the mechanisms of bioactive compounds regulating pyroptosis.

**Table 3:** Effects of bioactive compounds on cancer.

Bioactive Compound	Derived from	In Vivo/Vitro	Dose	Effect	Ref.
Dioscin	*Polygonatum zanlanscianense*, *Dioscorea* *nipponica* Makino, and *Dioscorea zingiberensis* Wright	In vitro	2, 4 μΜ in MG63 and U2OS 2.5, 5 μΜ in MNNG/HOS	Increase in caspase-3, cleaved GSDME-N Inhibition of cell proliferation Activation of JNK/p38 pathway	[54]
		In vivo	12, 24 mg/kg	Inhibition of OS proliferation	
DHA	Food	In vitro	50, 100, 200 μM	Increase in GSDMD, IL-1β, and caspase-1 Increase in HMGB1 Formation of membrane pore	[51]
Nobiletin	Citrus nobilis Lour., *C. aurantium* L., and *C. reticulata* Blanco.	In vitro	80 μM	Increase in IL-1β, IL-18, NLRP3, ASC, and cleaved caspase 1	[53]
Tanshinone IIA	*Salvia miltiorrhiz*	In vitro	2, 4, 8 μM	Decrease in cell proliferation Increase in caspase-3 and caspase-9 Increase in cleaved GSDMD, caspase-1, IL-1β, and IL-18	[55]
Curcumin	Turmeric and zedoary	In vitro	10, 20, 30 μM	Increase in GSDME-N-terminus Increase in ROS	[56]
Curcumin	Turmeric	In vitro	40 μM	Induction of caspase-1, HMGB1, IL-18, and IL-1β Decrease in NF-κB and toll-like receptors	[57]
Dihydroartemisinin	*Artemisia annua*	In vitro	5, 20, 40 μM	Increase in caspase-3, AIM2, GSDME, HMGB1IL-1β, and IL-18	[52]
		In vivo	4 mg/kg	Inhibition of breast xenograft tumors	

## 6. Bioactive Compounds Regulate Pyroptosis in Diabetes

Aberrant pyroptosis affects the initiation, promotion, and progression stages of diabetes and can be one of the most important factors that lead to common complications of diabetes [58]. It is also interesting to investigate how bioactive compounds ameliorate diabetes via pyroptosis (Table 4).

Epigallocatechin-3-gallate (EGCG), a polyphenol component of green tea, can improve glucose tolerance and alleviate NLRP3 inflammasome-mediated pyroptosis in a HFD-induced type 2 diabetes (T2D) mouse; further in vivo results were consistent with the in vitro data, which highlighted that EGCG inhibited pyroptosis by suppressing NLRP3 inflammasome, caspase-1 activation, and IL-1β secretion and blocked NLRP3-mediated ASC speck formation [59]. In another study, renal 4-hydroxynonenal, ROS, LC3-B/autophagy, IL-1β/pyroptosis, and caspase-3/apoptosis were declined by trehalose and guava juice in T2D rats [60]. In addition, curcumin was found to (i) improve diabetes/chronic cerebral hypoperfusion (CCH)-induced cognitive deficits, (ii) attenuate neuronal cell death, (iii) suppress neuroinflammation induced by microglial activation, and iv) reduce NLRP3-dependent pyroptosis [61]. Another report about diabetic atherosclerosis (DA) demonstrated that low doses of sinapic acid (≤50 mg/kg) inhibited the inflammasome assembly by decreasing NRLP3 and ASC and reducing caspase-1, IL-1β, and endothelin 1 (ET-1) in rats with DA; further in vitro results demonstrated that the pyroptosis process may be attributed to the downregulation of lncRNA-metastasis-associated lung adenocarcinoma transcript 1(MALAT1) exerted by synaptic acid in diabetic rats [62]. Ginsenoside Rb2 (Rb2) is one of the principal constituents of ginsenosides, with several beneficial effects. Recent research reported that Rb2 can regulate pyroptosis, improving insulin resistance (IR), by suppressing the expression of pyroptosis-related proteins, ASC, NLRP3, caspase-1, GSDMD, and IL-1β and declining the phosphorylation levels of p65 and IκBα both in vitro and in vivo, thus suggesting pyroptosis as a new promising therapeutic target for the diabetes treatment [63].

**Table 4:** Effect of bioactive compounds on diabetes.

Bioactive Compound	Derived from	In Vivo/Vitro	Dose	Effect	Ref.
Epigallocatechin-3-gallate	green tea	In vitro	20, 30, 40 μM	Decrease in IL-1β and NLRP3 inflammasome	[59]
		In vivo	50 mg/kg	Inhibition of NLRP3 inflammasome Improvement of glucose tolerance	
Quercetin-Rich Guava Juice and Trehalose	*Psidium guajav*	In vivo	Guava juice+ Trehalose: 4 + 2, 8 + 4, 20 + 1 mL/kg	Improvement of oral glucose tolerance test (OGTT), homeostasis model assessment of IR (HOMA-IR), and homeostasis model assessment of the function of β cell (HOMA-β) and plasma insulin Decrease in ROS, 4-hydroxynonenal, caspase-3, LC3-B, and IL-1β	[60]
Curcumin	*Curcuma longa*	In vitro	10 μM	Decrease in IL-18, ASC, GSDMD-N, cleaved-caspase-1, and NLRP3 Regulation of TREM2/TLR4/NF-κB signaling	[61]
		In vivo	50 mg/kg	Improvement of neuronal cell death and cognitive deficits Decrease in GSDMD-N, NLRP3, IL-18, cleaved-caspase-1, and ASC, Regulation of TREM2/TLR4/NF-κB signaling	
Sinapic acid	*Brassica alba* (L.) *Boiss*, *Brassica juncea* (L.) *Czern. et Coss*	In vitro	1 μM	Decrease in ASC, NRLP3, and caspase-1 Inhibition of lncRNA-MALAT1	[62]
		In vivo	5, 10, 50 mg/kg	Decrease in serum ET-1 and IL-1β Reduction of ASC, NRLP3, and caspase-1	
Ginsenoside Rb2 (Rb2)	*ginsenosides*	In vitro	50 μM	Improvement of IR Decrease in caspase-1, ASC, NLRP3, IL-1β, and GSDMD Reduction of the phosphorylation of p65 and IκBα	[63]
		In vivo	40 mg/kg	Improvement of body weight, fat accumulation, and IR Decrease in caspase-1, ASC, NLRP3, IL-1β, and GSDMD Reduction in the phosphorylation of p65 and IκBα	

## 7. Bioactive Compounds Regulate Pyroptosis in Tissue Injury

Tissue injury is usually accompanied by inflammation and programmed or non-programmed cell death. There are many kinds of tissue injury such as hepatic injury, kidney injury, lung injury, and brain injury. In recent years, numerous studies have reported that natural compounds have the potential to protect against tissue injury and the significance of pyroptosis in the therapeutic process has been highlighted (Table 5).

For example, in hepatic injury, DHA and arachidonic acid (AA) ameliorated liver function by decreasing the expression of the NLRP3 inflammasome complex, GSDMD, IL-1β, and IL-18 in LPS-induced Kupffer cells’ pyroptosis [64]. Similarly, Li et al. found that in hepatic ischemia reperfusion (I/R) injury rats, DHA displayed anti-pyroptosis activity by decreasing alanine aminotransferase (ALT), aspartate aminotransferase (AST), malondialdehyde (MDA), NLRP3 inflammasome, IL-1β, and IL-18, as well as increasing glutathione (GSH), catalase (CAT), and superoxide dismutase (SOD); on the other hand, the in vitro data suggested that DHA inhibited the hypoxia/restoration (H/R)-induced NLRP3 inflammasome assembly via the phosphoinositide 3-kinase (PI3K) pathway [65]. Moreover, vitamin D (VD) alleviated HFD-induced hepatic injury in rats both in vivo and in vitro in palmitic acid (PA)-treated or LPS-treated cells, and also reduced NLRP3 inflammasome activation and lipid accumulation in vivo and in vitro; the results also suggested that VD might attenuate non-alcoholic fatty liver disease (NAFLD) through pyroptosis because GSDMD-N fragment overexpression increased in cells with the activation of NLRP3 inflammasome in the VD group [66]. Cyclophosphamide (CP) is a common therapeutic drug for cancer. Long-term exposure can cause acute hepatotoxicity: ligustrazine (2, 3, 5, 6-tetramethylpyrazine, TMP) can reverse this situation by decreasing Txnip, p-NF-κB/NF-κB, and NLRP3 inflammasome-associated proteins (NLRP3, ACS, caspase-1, GSDMD-N, and IL-1β) and upregulating Trx in CP-treated mice, which was consistent with the in vitro experiment [67]. In addition, Yang et al. reported that limonin lightened liver injury by increasing the hepatic GSH amount and decreasing the serum ALT and AST levels and lactate dehydrogenase (LDH) release in LPS-treated mice. Additionally, an in vitro study showed that limonin blocked pyroptosis occurrence by decreasing ROS generation, NLRP3 inflammasome formation, caspase-1 activation, GSDMD cleavage, and IL-1β excretion, therefore declining the membrane pores [68].

Spinal cord injury (SCI) leads to many types of disabilities. The efficiency of clinical treatments is limited due to its complex pathophysiological process. A recent study showed that betulinic acid (BA) promoted functional recovery following SCI: BA was able to suppress the levels of NLRP3 inflammasome, GSDMD, IL-1β, and IL-18 in SCI rats. Further results reported that different types of cell death processes can be activated simultaneously; specifically, BA restored, following injury, the autophagy flux that promoted mitophagy to reduce ROS accumulation, and inhibited pyroptosis [69]. Another study on SCI showed that in rats, kaempferol reduced the activation of microglia-mediated oxidative stress, improved the recovery of hindlimb motor function, and finally, ameliorated tissue damage in vivo. Additionally, kaempferol not only inhibited the pyroptosis-related proteins (NLRP3, caspase-1 p10, ASC, and N-GSDMD) and the secretion of IL-18 and IL-1β, but also suppressed the phosphorylation of p38 mitogen-activated protein kinase (MAPK), c-Jun N-terminal kinase (JNK), and NF-κB p65 [70], while the relation between pyroptosis and the MAPK or NF-Κb pathway was not mentioned. Similarly, andrographolide (Andro) showed the inhibition of intracerebral hemorrhage (ICH)-induced secondary brain injury (SBI): specifically, GSDMD cleavage and caspase-1 activation were decreased in Andro- and edaravone-treated (positive control) groups and the two substances performed similar efficiency on ICH rats. Andro also inhibited pyroptosis by suppressing the interaction of ASC with Caspase-1 or the NLRP3 inflammasome [71]. Moreover, Zhang et al. observed, both in in vivo and in vitro experiments, that Gastrodin inhibited cerebral I/R injury by downregulating the NLRP3 inflammasome, cleaved caspase-1, and inflammatory factors (IL-1β and IL-18) [72]. In another in vivo model of ionizing radiation-induced intestinal injury, Li et al. found that *p*-coumaric acid (CA) suppressed the mRNA expression of pyroptosis genes (i.e., NLRP3, AIM2, and caspase-1) and improved the genes’ expression of the intestinal barrier [73]. Additionally, lycopene (Lyc) as a natural antioxidant has the potential to improve spleen injury: the supplementation of Lyc alleviated di (2-ethylhexyl) phthalate (DEHP)-induced inflammatory infiltration, suppressed the NLRP3 inflammasome, IL-18, IL-1β, and NF-κB, and increased GSDMD [74]. The overexpression of GSDMD indicated that Lyc could protect cell membrane integrity.

Curcumin has shown an anti-pyroptosis function in several diseases, including lung injury. Wang et al. demonstrated that curcumin reduced the levels of NLRP3, GSDMD-N, cleaved IL-1β, and cleaved caspase 1 and inhibited the expression of ASC and IL-1β in the CLP+200-Cur group in in vitro and in vivo models. This process may be achieved by preventing NLRP3 inflammasome-dependent pyroptosis activation through up-regulating Sirtuin 1 (SIRT1) [75].

**Table 5:** Effects of bioactive compounds on tissue injury.

Bioactive Compound	Derived from	In Vivo/Vitro	Dose	Effect	Ref.
DHA/AA	Food	In vitro	50 μM	Inhibition of IL-18, IL-1β, and NLRP3 inflammasome	[64]
		In vivo	50 mg/kg	Decrease in AL, AST, and LDH Inhibition of caspase-1 and NLRP3	
DHA	Food	In vitro	25 μM	Increase of cell viability Decrease in IL-1β, IL-18, NLRP3, caspase-1, and ASC Activation of PI3K/Akt pathway	[65]
		In vivo	300 mg/kg	Decrease in serum AST, ALT, IL-1β, and IL-18 Decrease in liver tissue MDA, caspase-1, ASC, and NLRP3 Increase in liver tissue GSH, SOD, and CAT	
Limonin	Citrus fruit	In vitro	10, 25, 50 μM	Decrease in ROS generation, LDH level, caspase-1, NLRP3, caspase-1, GSDMD, and IL-1β	[68]
		In vivo	50, 100 mg/kg	Decrease in serum ALT, AST, and LDH Increase in liver tissue GSH	
Betulinic acid	Chinese herbal	In vivo	20 mg/kg	Improvement of functional recovery Decrease in GSDMD, NLRP3, caspase-1, ASC, IL-1β, and IL-18 Induction of autophagy Decrease in ROS	[69]
Ligustrazine	*Rhizoma* Chuanxiong	In vitro	10, 20, 40 μM	Decrease in NLRP3, caspase-1, ACS, IL-1β, and GSDMD-N Inhibition of Txnip/Trx/NF-κB pathway Decrease in ALT, AST, MDA, and NO Decrease in ROS generation Increase in SOD, GSH, and CAT	[67]
		In vivo	20, 40 mg/kg	Improvement of liver structure, fibrosis, and cell death Decrease in NLRP3, ASC, caspase-1, GSDMD TNF-α, IL-6, and IL-1β Decrease in serum ALT and AST Decrease in liver MDA and NO Increase in liver SOD, GSH, and CAT Decrease in ROS generation Inhibition of Txnip/Trx/NF-κB pathway	
Andrographolide	*Andrographis paniculata*	In vitro	1, 3, 10 μM	Decrease in NLRP3 inflammasome Inhibition of NF-κB pathway Decrease in IL-1β, TNF-αIL-6, and LDH	[71]
		In vivo	0.5, 1, 2 mg/kg	Decrease in neuronal cell death and degeneration Improvement of neurobehavioral disorders and brain edema Decrease in TNF-α and IL-6 Inhibition of NLRP3 inflammasome	
*p*-coumaric acid	fruits, vegetables, and cereals	In vivo	50, 100, 200 mg/kg	Improvement of intestinal barrier morphology and apoptosis Increase in villus height and the ratio villus height/crypt depth Decrease in AIM2, NLRP3, and caspase-1	[72]
Lycopene	Fruits and vegetables with red color	In vivo	5 mg/kg	Decrease in NLRP3, caspase-1, IL-1β, IL-18, and ASC Increase in GSDMD Inhibition of NF-κB	[74]
Curcumin	*Curcuma longa*	In vitro	20, 40, 80 μM	Decrease in NLRP3, cleaved caspase 1, GSDMD, GSDMD-N, and cleaved IL-1β Inhibition of NF-κB and SIRT1	[75]
		In vivo	100, 200 mg/kg	Improvement of histopathological injury Decrease in myeloperoxidase (MPO), chemokine (C-C motif) ligand 7 (CCL7), IL-6, and TNF-α Inhibition of SIRT1	
Kaempferol	tea, kale, broccoli, cabbage, grapefruit	In vitro	25, 50, 100 μM	Decrease in microglia activation, ROS, and NADPH oxidase 4 Inhibition of MAPK pathway and NF-κB p65 translocation Decrease in NLRP3 caspase-1 p10, ASC, N-GSDMD, IL-18, and IL-1β	[70]
		In vivo	25, 50, 100 mg/kg	Improvement of tissue damage and hindlimb motor function Decrease in oxidative stress and microglia activation	
Gastrodin	Tianma	In vitro	5 μg/mL	Inhibition of cleaved caspase-1, NLRP3, IL-1β, and IL-18 Modulation of lncRNA NEAT1/miR-22-3p pathway	[72]
		In vivo	50 mg/kg	Improvement of neurological scores Decrease in the area of cerebral infarction Inhibition of cleaved caspase-1, NLRP3, IL-1β, and IL-18	
Vitamin D	Food	In vitro	10^−8^, 10^−7^, 10^−6^ mol/L	Improvement and lipid accumulation Decrease in NLRP3 inflammasome, GSDMD-N, and IL-1β	[66]
		In vivo	5 μg/kg	Decrease in NLRP3 inflammasome, IL-1β, and IL-18 Decrease in serum AST, ALT, and triglyceride (TG) Improvement of gut microbiota dysbiosis	

## 8. Bioactive Compounds Regulate Pyroptosis in Heart Disease

Many bioactive compounds exert a cardiac protection function. When pyroptosis was discovered, its mechanism was widely reported, and there is no doubt that pyroptosis plays an important role in heart disease (Table 6).

For example, pinocembrin (PCB), a flavonoid present in the propolis and rhizomes of *Boesenbergia pandurate,* exerts different biological activities. Gu et al. reported that PCB administration improved doxorubicin (DOX)-induced cardiac dysfunction, both in in vitro and in vivo models, by inhibiting pyroptosis [76]. Interestingly, the results from an in vitro study suggested that myocardial pyroptosis inhibition by PCB can be impaired by a decrease in Sirt3 and Nrf2 levels, suggesting that pyroptosis was at least partly suppressed by the Nrf2/Sirt3 signal pathway [76]. Similarly, Li et al. found that sweroside pretreatment, both in vivo and in vitro, protected against myocardial IR injury by suppressing NLRP3 inflammasome-mediated pyroptosis and oxidative stress partly through the modulation of the Keap1/Nrf2 pathway [77]. In addition, astragaloside IV (AS-IV), the *Astragalus membranaceus* major bioactive component, was able to reduce myocardial fibrosis and myocardial hypertrophy, which may be attributed to decreased ROS levels and the suppression of the NLRP3/Caspase-1/GSDMD pyroptosis pathway after AS-IV treatment [78]. Another interesting piece of research in rats showed that Rb1 not only improved myocardial damage induced by aconitine, but enhanced the contractile function and field potential of cardiomyocytes by modulating calcium channels and also decreased myocardial cell damage by constraining ventricular myocyte calcium transients [79]. According to previous research, the decrease in calcium overload may reduce cell pyroptosis and, consequently, the damage to the myocardium [80], suggesting that maintaining calcium homeostasis may contribute to suppressing the inflammatory response associated with pyroptosis in the heart [79].

**Table 6:** Effect of bioactive compounds on heart disease.

Bioactive Compound	Derived from	In Vivo/Vitro	Dose	Effect	Ref.
Pinocembrin	*Boesenbergia pandurate*	In vitro	1 μM	Inhibition of NLRP3 inflammasome, Nrf2, and Sirt3	[76]
		In vivo	5 mg/kg	Improvement of cardiac fibrosis and cardiac function Reduction in left ventricular internal dimension in diastole (LVIDd), left ventricular internal dimension in systole (LVIDs), left ventricular fractional shortening (LVFS), and left ventricular ejection fraction (LVEF), Decrease in CK-MB, LDH, IL-18, and IL-1β	
Astragaloside IV	*Astragalus*	In vitro	100 μM	Decrease in NLRP3, GSDMD-N, cleaved caspase-1, cleaved IL-18, and cleaved IL-1β	[78]
		In vivo	40 mg/kg	Improvement of poor ventricular remodeling, myocardial fibrosis, and myocardial hypertrophy Decrease in ROS, NLRP3 inflammasome, and GSDMD Decrease in macrophages and neutrophils	
Sweroside	*Swertia pseudochinensis* Hara	In vitro	50 μM	Reduction in LDH, CK-MB, MDA, and ROS Increase in SOD and glutathione peroxidase (GSH-Px) Decrease in NLRP3, ASC, IL-1β, and cleaved caspase-1 Inhibition of Keap1 and Nrf2	[77]
		In vivo	20, 50, 100 mg/kg	Reduction in infarct size Improvement of cardiac function	
Ginsenoside Rb1	ginseng, *panax notoginseng*, and *American ginseng*	In vitro	25, 50, 100 μM	Inhibition of calcium transients Improvement of contractile function and field potential	[79]
		In vivo	10, 20, 40 mg/kg	Inhibition of apoptosis and pyroptosis Improvement of myocardial damage	

## 9. Bioactive Compounds Regulate Pyroptosis in Other Diseases

The effects of bioactive compounds on pyroptosis have also been studied in other human diseases (Table 7).

Nephrolithiasis is a common urinary disease that presents a high recurrence rate of secondary stone formation. Vitexin was found to alleviate crystal deposition and kidney tissue injury in vivo in mice by decreasing NLRP3 inflammasome levels, cleaved caspase-1, GSDMD, and IL-1β and repressing apoptosis as well as the expression of osteopontin (OPN) and CD44. Additionally, both in HK-2 cells and THP-1 macrophages treated with calcium oxalate monohydrate (COM) to create a model of crystal-induced cell injury, vitexin-inhibited pyroptosis blocked the Wnt/β-catenin pathway and suppressed the expression of IL-1β and tumor necrosis factor-α (TNF-α) [81].

Baeckein E (BF-2) from *Baeckea frutescens* L. can be a beneficial bioactive compound for gout: in macrophages, it can suppress IL-1β secretion and cell pyroptosis as well as, in a gout mouse model, inhibit the activation of the NLRP3 inflammasome and reduce ankle swelling; its property of inhibiting NLRP3 inflammasome activation may be attributed to the MAPK/NF-κB pathways’ suppression [82].

Atherosclerosis is a chronic inflammatory disorder and seems to involve pyroptosis [83,84]. The natural flavonoid dihydromyricetin (DHM) was shown to inhibit PA-induced pyroptosis by decreasing IL-1β and LDH secretion, preserving cell membrane integrity and declining IL-1β maturation and caspase-1 cleavage, whereas Nrf2 knockdown by siRNA revoked the DHM inhibitory effects, indicating the partial involvement of the Nrf2 signaling pathway in the DHM-induced pyroptosis improvement of vascular endothelial cells [85]. In another research on Alzheimer’s Disease (AD), schisandrin (SCH) was proven to improve in AD mice cognitive impairment by inhibiting neural pyroptosis and apoptosis induced by the NLRP1 inflammasome, even though the profound mechanism is unknown [86]. In addition, isoliquiritin, derived from *Glycyrrhiza uralensis*, showed potential antidepressant properties by inhibiting pyroptosis though the miRNA-27a/spleen tyrosine kinase (SYK)/NF-κB pathway [87]. Specifically, low miRNA-27a expression was evaluated in rodent models and depressed patients without isoliquiritin treatment; however, isoliquiritin treatment not only increased miRNA-27a expression, but also decreased SYK, p-NF-κB, and pyroptosis-related protein levels both in in vivo and in vitro models where the miRNA-27a inhibitor reversed the isoliquiritin therapeutic efficacy [87].

Finally, Gowda et al. assessed the effect of glycyrrhizin, a natural compound derived from licorice, on COVID-19 [88]. The results showed that glycyrrhizin alleviated the activation of macrophages and viral-proteins-induced pyroptosis, reduced the release of IL-1β, IL-6, and IL-8, and decreased the levels of ferritin from macrophages cultured in conditioned media from lung cells expressing Orf3a and SARS-CoV-2 S-RBD; additionally, glycyrrhizin repressed SARS-CoV-2 replication in Vero E6 cells [88].

**Table 7:** Effect of bioactive compounds on other diseases.

Bioactive Compound	Disease	In Vivo/Vitro	Dose	Effect	Ref.
Baeckein E	Gout	In vitro	0.4, 0.8, 1.6 μM	Decrease in IL-1β and NLRP3 inflammasome Improvement of mitochondrial damage Suppression of MAPK/NF-κB pathway	[82]
		In vivo	12.5, 50 mg/kg	Suppression of NLRP3 inflammasome and ankle swelling Inhibition of NF-κB	
Glycyrrhizin	COVID-19	In vitro	1 mM	Decrease in IL-1β, IL-6, IL-8, and ferritin SARS-CoV-2 replication inhibition	[88]
Dihydromyricetin	Atherosclerosis	In vitro	300 μM	Decrease in LDH, IL-1β, and caspase-1 cleavage Suppression of intracellular and mitochondrial ROS Activation of the Nrf2	[85]
Vitexin	Nephrolithiasis	In vitro	10, 20 mg/L	Decrease in LDH Inhibition of pyroptosis-related proteins Increase in pan-cytokeratin (Pan-ck) expression Decrease in Vimentin and alpha-smooth muscle actin (α-SMA) expression Downregulation of Wnt/β-catenin pathway Suppression of TNF-α and IL-1β	[81]
		In vivo	10, 20 mg/kg	Improvement of crystal deposition and kidney tissue injury Decrease in MDA Increase in SOD, GSH, and CAT Reduction in GSDMD, NLRP3, cleaved caspase-1, and IL-1β Suppression of CD44 and OPN expression Improvement of MCP-1 expression and F4/80-positive macrophage infiltration	
Schisandrin	Alzheimer’s disease	In vitro	10 μM	Suppression of neuronal apoptosis and pyroptosis	[86]
		In vivo	2 mg/kg	Improvement of cognitive impairment Inhibition of Aβ production Suppression of neuronal apoptosis and pyroptosis Inhibition of NLRP1, ASC, caspase-1, IL-18, and IL-1β	
Isoliquiritin	Depression	In vitro	50 μM	Increase in miRNA-27a Decrease in p-NF-κB, SYK, NLRP3, cleaved caspase-1, IL-1β, and GSDMD-N	[87]
		In vivo	10, 30 mg/kg	Improvement of sucrose preference and neuronal cells disorder Increase in miRNA-27a and NeuN Decrease in p-NF-κB and SYK Inhibition of GSDMD-N, NLRP3 inflammasome, IL-1β, and TNF-α	
		Clinical	No treatment	Decrease in miRNA-27a	

## 10. Conclusions

Bioactive compounds can improve human diseases through various molecular mechanisms. The current evidence indicates that bioactive compounds regulate pyroptosis mainly via the NLRP3/caspase-1/GSDMD pathway, while NLRP1/caspase-1, AIM2/caspase-3/GSDME, and caspase-11/GSDMD, also participate in this process. In addition, pyroptosis, accompanied by the activation or inactivation of many signal pathways (PI3K/Akt, AMPK, PKA, NF-κB, MAPK, Keap1/Nrf2, etc.), exerts different functions in diverse diseases, which determine if the bioactive compounds may promote or suppress it. For example, bioactive compounds induce pyroptosis in cancer, while they inhibit it in almost all other diseases, highlighting the precise regulation of bioactive compounds on pathological processes. From this review, bioactive compounds seem to have a powerful potential to be the candidate drug for pyroptosis modulation in many common diseases, but more clinical studies are needed to confirm efficient doses for humans. The mechanisms of pyroptosis regulation by bioactive compounds are still unclear even if a variety of molecular pathways are reported to have a close relationship with it. Nevertheless, the current evidence shows the strong anti-pyroptosis ability of bioactive compounds in both in vitro and in vivo models, even if no clinical research has yet to support pyroptosis as a significant target for the development of bioactive compound-based drugs in disease therapy.

## Data Availability

No new data were created or analyzed in this study. Data sharing is not applicable to this article.
